# IL-32θ inhibits monocytic differentiation of leukemia cells by attenuating expression of transcription factor PU.1

**DOI:** 10.18632/oncotarget.3013

**Published:** 2015-01-23

**Authors:** Man Sub Kim, Jeong-Woo Kang, Yun Sun Park, Dong Hun Lee, Yesol Bak, Taeho Kwon, Do-Young Yoon

**Affiliations:** ^1^ Department of Bioscience and Biotechnology, Bio/Molecular Informatics Center, Konkuk University, Seoul, Republic of Korea

**Keywords:** myeloid differentiation, Interleukin-32, PU.1, C/EBPα

## Abstract

PU.1 is a key transcription factor regulating the myeloid differentiation. PU.1-induced monocytic differentiation into macrophage is also important for blood cancer development. Therefore, we chose THP-1 monocytic leukemia cells to investigate the function of a recently discovered IL-32θ. Genetic analyses identified differences in the sequences of *IL-32θ* and *IL-32β*. Using previously established cell lines that stably express IL-32θ and IL-32β and cell lines transiently expressing IL-32θ, we observed that expression of IL-32θ inhibited phorbol 12-myristate 13-acetate (PMA)-induced monocytic differentiation in both THP-1 and HL-60 cells. IL-32θ also suppressed expression of the macrophage cell surface markers, CD11b, CD18, and CD36. Interestingly, expression of IL-32β or IL-32θ had no effect on the expression levels of cell cycle related factors. As a result, we concluded that these isoforms did not contribute to PMA-induced cell cycle arrest. IL-32θ was found to modulate expression of PU.1, a transcription factor necessary for myeloid lineage commitment. Transient expression of PU.1 in THP-1/IL-32θ cells rescued the observed differentiation defect. Additionally, transient expression of both CCAAT-enhancer-binding protein α (C/EBPα) and PU.1 in THP-1/IL-32θ cells exhibited synergistic effects in rescuing the differentiation defect. These observations indicate that intracellular IL-32θ inhibits the differentiation of monocytes into macrophages by attenuating PU.1 expression.

## INTRODUCTION

Myeloid cells as pivotal effectors of innate immune reaction are important regulators of adaptive immunity [1]. It has been known that the differentiation and activation of myeloid cells involve quantitative regulation of essential transcription factors, including PU.1, interferon regulatory transcription factor (IRF) family, CCAAT-enhancer-binding proteins (C/EBPs) and runt-related transcription factor 1 (RUNX1) [2–3]. Dysregulation of these key transcription factors induce and affect blood cancer development as well as innate immune response [4]. Therefore, disorder of myeloid differentiation is a typical feature of acute myeloid leukemia (AML) [5]. Monocytes involved in myeloid lineage commitment are derived from pluripotent hematopoietic stem cells, and can differentiate into macrophages or dendritic cells depending on external stimuli [6–7]. Monocytes identify ongoing viral or bacterial infections, and then infiltrate the infected tissue where they differentiate into the appropriate effector cell type [8].

E26 transformation-specific (Ets) family which is a large group of transcription factors was transduced for the first time by leukemia virus, E26 [9]. PU.1 is a member of the ETS-family of transcription factors, has ETS domain which can recognize and interact ‘GGAA’ and ‘GGAT’ DNA motif, respectively [10]. The cellular function of PU.1 as a transcription factor is mainly involved in myeloid and B cell lineage development [11]. Therefore, PU.1 expression is tightly regulated in multiple hematopoietic lineages [12]. Disruption of PU.1 in mice led to lack macrophages, neutrophils, and B and T cells [13–14]. PU.1 also plays a key role as tumor suppressor for B cell malignancies [15] and classical Hodgkin lymphoma cells [16]. Indeed, an excess of PU.1 expression was found to block differentiation during development of myeloid and erythroid lineages by interacting with the transcription factors GATA-1 and GATA-2 [17–18].

IL-32 was characterized as a proinflammatory cytokine because it was expressed in the lesions of patients with rheumatoid arthritis [19]. Six isoforms of IL-32, generated by alternative splicing of the IL-32 mRNA, were previously shown to exist. Recently, however, three additional isoforms, IL-32η, IL-32θ, and IL-32s, were characterized [20]. IL-32γ, which can be spliced into IL-32β by post-translational modification, is the most highly expressed of the isoforms, as observed in inflammatory diseases and cancers [21]. As a result, IL-32β, rather than other isoforms, is commonly detected at higher levels in various immortalized cell lines [22–23]. IL-32α and IL-32γ can induce expression of other proinflammatory cytokines, including IL-6, IL-8 [24–26]. Although IL-32 is a crucial component of the immune response, previous studies on the functions of IL-32 have primarily concentrated on four of the isoforms: IL-32α, IL-32β, IL-32δ, and IL-32γ [27–28]. IL-32θ is an isoform that was recently identified in lipopolysaccharide (LPS)-differentiated dendritic cells, purified from the human periphery [20]. As a result, the function of this isoform has yet to be characterized.

Recent studies indicated that IL-32 modulates the differentiation of monocytic cells and regulates the production of inflammatory cytokines. Here, we demonstrate that the newly discovered isoform, IL-32θ, suppresses monocyte differentiation by regulating the expression of the PU.1 transcription factor.

## RESULTS

### Elucidation of the IL-32θ and IL-32β coding sequences

**Figure 1:**
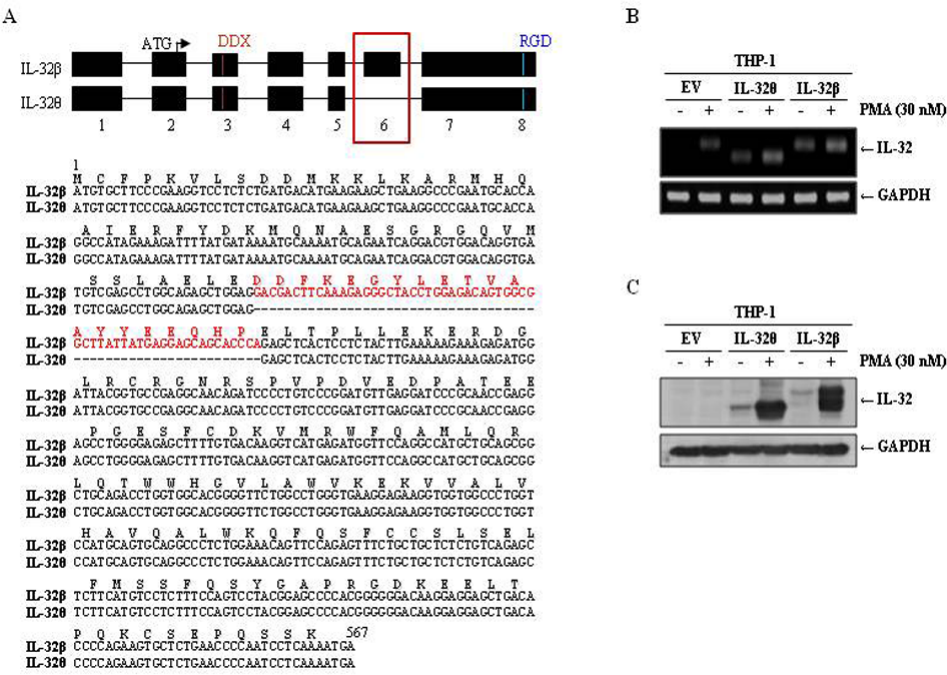
Comparison of the coding sequences and expression levels of IL-32θ and IL-32β **(A)** The schematic diagram of the coding sequences of IL-32θ and IL-32β. The difference of exon 6 between IL-32β and IL-32θ was indicated with red box and presented with amino acid and mRNA sequence. We established THP-1 cell lines expressing IL-32β or IL-32θ. Cells were treated with 30 nM PMA for 72 h and expression levels were measured by RT-PCR using IL-32-specific primers **(B)** and by Western blot using an IL-32-specific antibody **(C)**. GAPDH was used as a control in both experiments, and IL-32 expression was normalized to GAPDH expression.

IL-32β is abundantly expressed in various tissues. It is also highly expressed in cancerous tissues and regions where inflammation is present. Conversely, expression of IL-32θ has been detected in dendritic cells derived from human peripheral blood monocytic cells (PBMCs). As a result, the functions of this isotype are thought to be limited. Because monocytes express IL-32β endogenously, we chose monocytes to compare the functions of IL-32θ to IL-32β in a monocytic line. We first analyzed the coding sequences of each isoform and determined that the IL-32θ mRNA lacks exon 6 (GenBank, accession number FJ985780), which is present in that of IL-32β (Figure 1A). The differences of both IL-32 isoforms are distinguished from alternative mRNA splicing after transcription of IL-32 mRNA [21]. The IL-32 coding sequences were then cloned into mammalian expression vectors and used to establish THP-1 myelomonocytic cell lines stably expressing the IL-32 isoforms. THP-1 cells were chosen because they have been used previously in a monocyte-to-macrophage differentiation model [33–35]. The resulting strains THP-1/IL-32θ and THP-1/IL-32β were subjected to RT-PCR and immunoblot analyses to assess IL-32 production in the presence or absence of phorbol 12-myristate 13-acetate (PMA) treatment, which has been used to induce monocyte differentiation into macrophage-like cells. Interestingly, the expression levels of both IL-32 isoforms were dramatically increased in the populations treated with 30 nM of PMA for 3 days compared to the untreated populations (Figure 1B and 1C).

### PMA-induced differentiation of THP-1 monocytes into macrophages is regulated by IL-32θ

**Figure 2:**
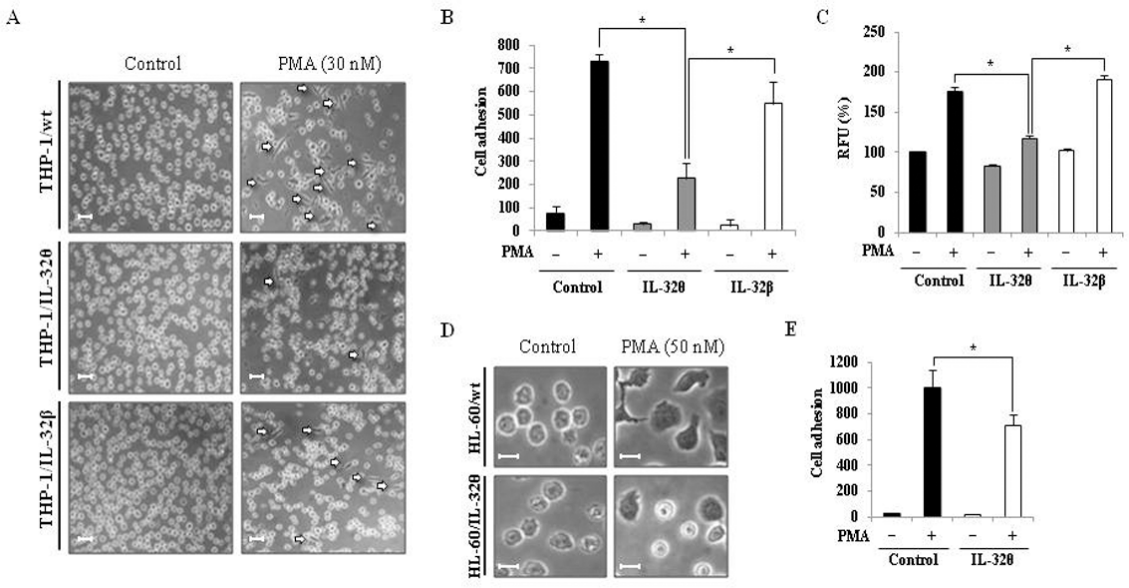
Effects of IL-32θ and IL-32β expression on cell morphology and adhesion capacity in THP-1 cell lines THP-1/IL-32θ and THP-1/IL-32β cells were stimulated with 30 nM PMA for 72 h and **(A)** morphological changes were assessed by phase-contrast microscopy (100×) and **(B)** by quantifying cell adherence to culture dishes. Scale bars represent 20 μm. **(C)** Under identical conditions, cell adhesion capacity was measured using the leukocyte-endothelium adhesion assay kit and HUVEC endothelial cells. Treatment with 50 nM of PMA for 72 h was optimal for inducing differentiation of HL-60 cells. HL-60 cells were transfected with 1 μg pcDNA3.1+ empty vector or pcDNA 3.1+-6 × Myc-IL-32θ and incubated overnight. After PMA treatment for 72 h, HL-60 cell morphologies were examined by phase-contrast microscopy (200×) **(D)** and cell adherence to culture dishes was quantified **(E)**. Scale bars represent 10 μm. Data are presented as mean ± standard error of mean (*n* = 3). *p < 0.05. THP-1/IL-32θ cells versus THP-1/wt or THP-1/IL-32β cells, after PMA treatment.

Upon injury, undifferentiated monocytes, which are generally localized within the blood, infiltrate the wound site and differentiate into macrophages or dendritic cells [36]. As a result, the specific morphological changes that occur during differentiation of a monocyte into macrophage-like cell, are crucial to the acute innate immune response. We previously reported that the IL-32α isoform inhibited both PMA-induced morphological changes and CD18/PU.1 expression in human monocytes [31]. This finding was evidenced on the interrelationship between IL-32 and monocytic differentiation because PU.1 plays a crucial role in myeloid lineage development [37]. Thus, we predicted that other isoforms may influence the differentiation of monocytes. To address this possibility, differentiation was first assessed by examining the morphology of THP-1/wt, THP-1/IL-32θ, and THP-1/IL-32β cells and quantifying adherence of differentiated cells to culture plates after stimulation with 30 nM PMA. From these analyses, we determined that the level of differentiation in the THP-1/IL-32θ population was less than 50% of that observed in the THP-1/wt and THP-1/IL-32β cells after PMA stimulation (Figure 2A and 2B). In addition, we examined the ability of these cell lines to adhere to vascular endothelium, using cultured HUVEC endothelial cells. Similar to results observed in the culture dishes, adhesion of THP-1/IL-32θ cells to HUVEC cells was significantly reduced compared to the adhesion of THP-1/wt and THP-1/IL-32β cells (Figure 2C). To then assess the relevance of IL-32θ expression in another cell line, we transiently transfected HL-60 mononuclear cells with the IL-32θ expression vector and observed any morphological changes that occurred upon PMA treatment. Compared to the wild type control, morphological changes were partially impaired in the HL-60/IL-32θ cell population (Figure 2D). Furthermore, the amount of PMA treated HL-60/IL-32θ cells that adhered to a culture dish was significantly lesser than the amount of adherent wild type cells (Figure 2E). These findings indicate that increased levels of intracellular IL-32θ, but not IL-32β, attenuate the differentiation of monocytes into macrophage after PMA stimulation.

### IL-32θ attenuates expression of monocyte/macrophage differentiation markers

**Figure 3:**
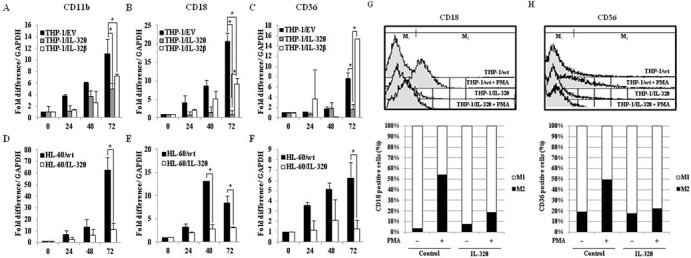
Expression of macrophage-specific cell surface markers in IL-32θ- and IL-32β-expressing cells after PMA stimulation THP-1/wt, THP-1/IL-32θ, and IL-32β cell lines were treated with 30 nM PMA for 72 h and CD11b **(A)** CD18 **(B)** and CD36 **(C)** mRNA expression levels were measured by qRT-PCR. HL-60 cells were transfected with 1 μg IL-32θ expression vector and incubated overnight. Cells were then treated with 50 nM PMA for 72 h and CD11b **(D)**, CD18 **(E)**, and CD36 **(F)** mRNA expression levels were measured by qRT-PCR. Under identical conditions, THP-1/wt and THP-1/IL-32θ cells were harvested and fixed with 100% acetone. Cells were then labeled with marker-specific primary antibodies and FITC-conjugated secondary antibodies. The expression levels of CD18 **(G)** and CD36 **(H)** were measured by FACs analysis. Data are presented as mean ± standard error of mean (*n* = 3). *p < 0.05. THP-1/IL-32θ cells versus THP-1/wt or THP-1/IL-32β cells, after PMA treatment.

To further investigate whether expression of IL-32θ inhibits differentiation of monocytes into macrophages, we measured the expression of the macrophage-specific differentiation markers CD11b, CD18, and CD36 by qRT-PCR analysis. Expression of the macrophage-1 antigen (Mac-1, CD11b/CD18) was dramatically reduced in THP-1/IL-32θ and HL-60/IL-32θ cells compared to wild type cells (Figure 3A, 3B, 3D and 3E). These expression patterns were similar to those reported for IL-32α [31]. In addition, CD36, which is highly expressed during differentiation into macrophages, was suppressed in the IL-32θ cell lines (Figure 3C and 3F). The expression patterns of the differentiation markers were erratic in the THP-1/IL-32β cells. To confirm the expression levels of the cell surface markers and detect differentiation into macrophages, we performed FACS analysis using marker-specific primary antibodies and FITC-conjugated secondary antibodies. The numbers of CD18 and CD36 positive cells (gated in M_2_) were greater in the PMA-treated THP-1/wt population than in the non-treated cells. However, expression of these markers was nearly identical in the treated and untreated THP-1/IL-32θ populations (Figure 3G and 3H). These findings suggest that intracellular IL-32θ inhibits expression of macrophage specific markers during PMA-induced monocyte differentiation into macrophages. Conversely, IL-32β appears to be irrelevant to this process.

### Ectopic expression of IL-32θ decreases PMA-induced monocyte differentiation

**Figure 4:**
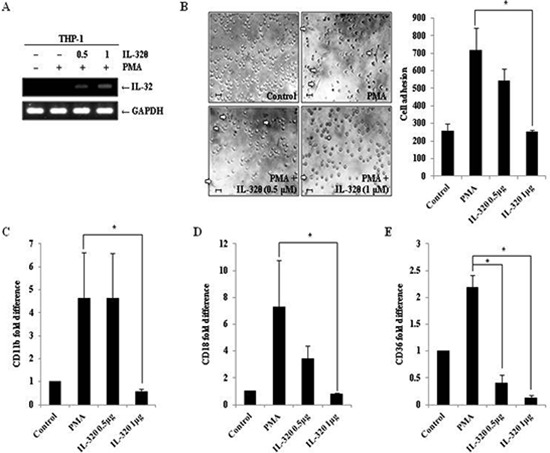
Ectopic expression of IL-32θ inhibits differentiation of THP-1 cells **(A)** THP-1 cells were transfected with empty pcDNA3.1+ vector or the indicated amount of pcDNA3.1+-IL-32θ-6 × Myc and incubated overnight. Cells were then treated with 30 nM PMA for 72 h and RT-PCR was used to confirm IL-32 expression in the transfected cells. **(B)** The morphologies of cells ectopically expressing IL-32θ were assessed by phase-contrast microscopy (100×). Scale bars represent 10 μm. After transfection and PMA stimulation, undifferentiated cells (non-adhering cells) were washed out of the plates with PBS and adherent, differentiated cells were fixed, stained, and quantified. To measure expression of the macrophage-specific markers CD11b **(C)**, CD18 **(D)**, and CD36 **(E)**, cells were treated and harvested, as previously described, and qRT-PCR was performed using marker-specific primers. Data are presented as mean ± standard error of mean (*n* = 3). *p < 0.05. THP-1 cells versus THP-1 cells with ectopic expression of IL-32θ, after PMA treatment.

To confirm whether IL-32θ inhibits monocytic differentiation, wild type THP-1 cells were transfected with an IL-32θ expressing vector. Expression of IL-32θ by the transfected cells was confirmed by RT-PCR (Figure 4A). Transfected THP-1 cells were stimulated with 30 nM of PMA and morphological changes were observed. The number of differentiated cells was reduced by IL-32θ expression in a transfection dose-dependent manner (Figure 4B). To further assess the effect of intracellular IL-32θ on the expression of macrophage-specific markers, the mRNA levels of CD11b, CD18, and CD36 were measured in cells transiently expressing IL-32θ. Consistent with results obtained from the stably expressing cell lines, the expression levels of all three macrophage-specific markers were decreased in the cells transfected with the IL-32θ construct compared to wild type (Figure 4C–4E). These data supported the conclusion that inhibition of monocytic differentiation was due to the intracellular IL-32θ expression.

### Expression of IL-32θ or IL-32β does not affect PMA-induced cell cycle arrest in G_0_/G_1_ phase

**Figure 5:**
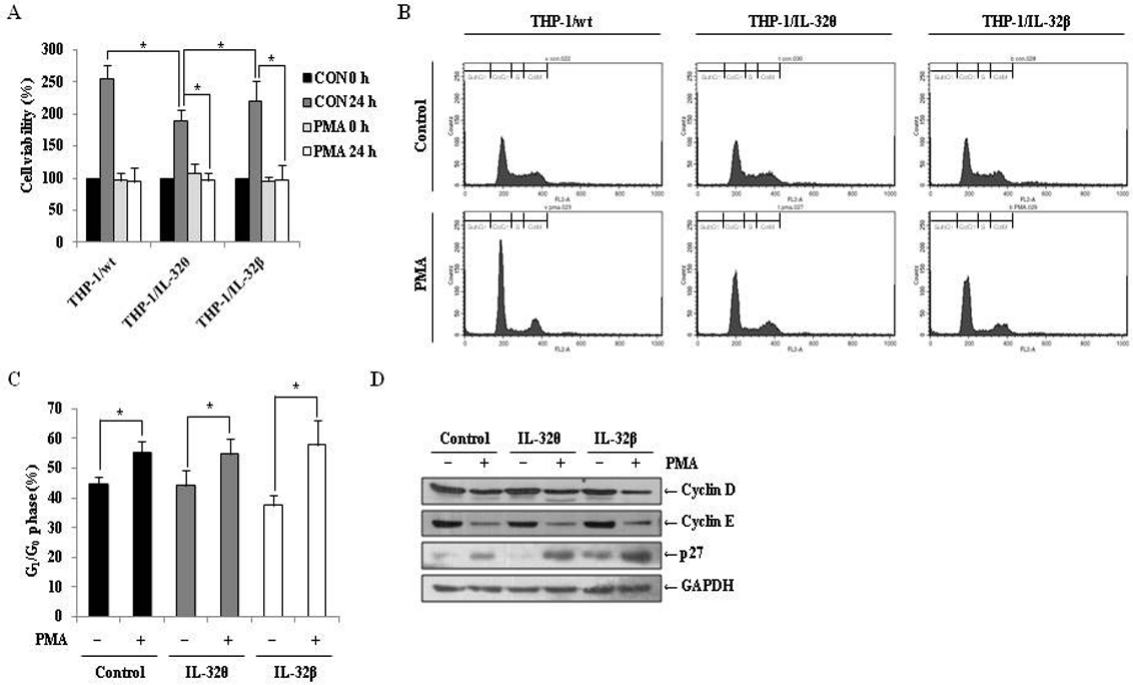
Effects of IL-32θ and IL-32β on PMA-mediated cell cycle arrest in THP-1 cells **(A)** THP-1/Wt, THP-1/IL-32θ, and THP-1/IL-32β cells were synchronized by starvation overnight, and then seeded onto 96-well plates and treated with 30 nM PMA in complete medium for 72 h. Cell proliferation was measured using an MTS assay. **(B)** To determine the cell cycle phase at which proliferation of IL-32θ and IL-32β cells was halted, cells were stained with propidium iodide (PI) containing RNase A, and the population of each cell cycle phase was measured by flow cytometry. **(C)** The G_0_/G_1_ cell cycle phase distribution is summarized in the bar graphs. Y-axes are the proportion of counted events in an indicated phase. Data are presented as mean ± standard error of mean (*n* = 3). *p < 0.05. Untreated cells versus 30 nM PMA treated cells in each cell lines. **(D)** Expression levels of the G_0_/G_1_ phase-related factors cyclin D, cyclin E, and p27 were assessed by Western blot analysis, using specific antibodies. GAPDH was used as a loading control.

PMA-induced differentiation of monocytes into macrophage is accompanied by cell cycle arrest [38]. Therefore, we quantified the number of viable THP-1/wt, THP-1/IL-32θ, and THP-1/IL-32β cells after PMA treatment and compared them to untreated populations. In the wild type and IL-32β-expressing cells, PMA treatment resulted in a significant reduction in the number of viable cells after 24 h, compared to the untreated control group. Interestingly, similar results were obtained in the IL-32θ expressing cells (Figure 5A), even though our results demonstrated that monocytic differentiation was not induced in this population (Figure 2). These results indicate that cell proliferation was halted in each of the three cell types after PMA treatment. To determine the phase of the cell cycle at which proliferation was interrupted, treated cells were stained with propidium iodide (PI), and populations were quantified by FACs analysis. As depicted in Figure 5B and 5C, the populations from each cell line were concentrated in the G_0_/G_1_ phase. Furthermore, immunoblot analyses detected decreased levels of the cell cycle regulatory proteins, cyclin D and E, and increased expression of p27, which regulates cell cycle arrest associated with cyclin E, in each of the PMA-treaded populations, in comparison to the untreated control groups (Figure 5D). These findings indicate that while IL-32θ and IL-32β were not the cause of the cell cycle arrest after PMA treatment, IL-32θ may regulate cell proliferation regardless of PMA treatment.

### IL-32θ attenuates expression of PU.1, a transcription factor necessary for differentiation of monocytes into macrophages

**Figure 6:**
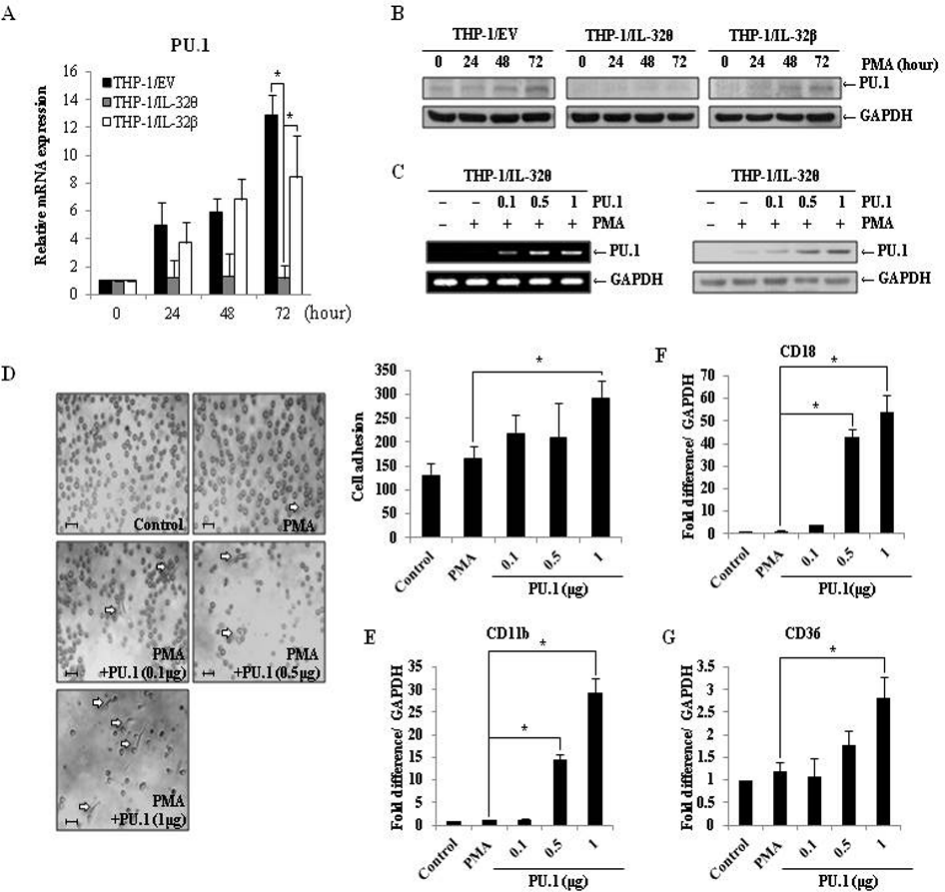
Expression of PU.1 rescues the IL-32θ-induced differentiation defect **(A)** THP-1/wt, THP-1/IL-32θ and THP-1/IL-32β cells were treated with 30 nM PMA for 72 h. Expression levels of PU.1 were measured by qRT-PCR, using PU.1-specific primers. Data are presented as mean ± standard error of mean (*n* = 3). *p < 0.05. THP-1/IL-32θ cells versus THP-1/wt or THP-1/IL-32β cells after PMA treatment. **(B)** PU.1 expression was confirmed by Western blot. Cells were treated and harvested as described above, and protein was detected using an α-PU.1 antibody. Detection of GAPDH was used as an internal control. To assess PU.1-mediated restoration of monocyte differentiation, THP-1/IL-32θ cells were transfected with the indicated amount of empty pcDNA3.1+ vector or the indicated amount of pCDNA 3.1+-PU.1-HA. **(C)** Cells were treated with 30 nM PMA for 72 h, and the expression levels of PU.1 were confirmed by RT-PCR and Western blot analyses. **(D)** The morphology of transfected cells was observed by phase-contrast microscopy (200×). Scale bars represent 20 μm. Undifferentiated cells were washed out twice with PBS and adherent cells were stained with Diff-Quick solution. Adhesion levels were evaluated by quantifying the number of stained cells from three randomly selected fields. To measure expression levels of CD11b **(E)**, CD18 **(F)**, and CD36 **(G)**, cells were treated and harvested as described above, and qRT-PCR was used for analysis. Data are presented as mean ± standard error of mean (*n* = 3). *p < 0.05. THP-1/IL-32θ cells versus cells transfected with indicated dose of PU.1, after PMA treatment.

The regulatory role of PU.1 during myeloid lineage development has been widely studied [39]. Using qRT-PCR and immunoblot approaches, we found that expression of PU.1 was suppressed in THP-1/IL-32θ cells compared to the THP-1/wt and THP-1/IL-32β cells after PMA treatment (Figure 6A and 6B). To assess whether PU.1 could rescue the observed monocyte differentiation defect, PU.1 was transiently expressed in THP-1/IL-32θ cells, in which endogenous PU.1 expression is inhibited by IL-32θ. For these experiments, the dose-dependent effects of PU.1 were also assessed by transfecting THP-1/IL-32θ cells with 0.1, 0.5, or 1 μg of pcDNA-3.1+-PU.1-HA (Figure 6C). Interestingly, the morphology and adhesion levels of cells transfected with 1 μg of the PU.1 vector were similar to those observed in the wild type macrophage-like population (Figure 6D). Furthermore, the expression levels of the macrophage-specific markers, CD11b, CD18, CD36, were increased in cells transiently expressing PU.1, in a dose-dependent manner (Figure 6E, 6G, 6F). These findings indicate that PU.1 is a key regulatory component that can restore differentiation in THP-1 by inhibiting the effects of IL-32θ.

### C/EBPα expression leads to additive effect with PU.1 on the restoration of monocytic differentiation

**Figure 7:**
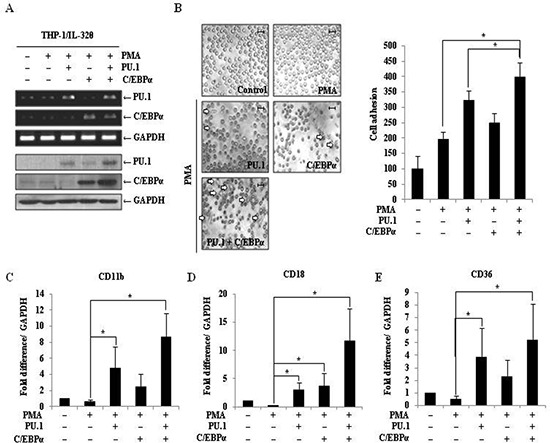
Effect of PU.1 and/or C/EBPα expression on monocyte differentiation in THP-1/IL-32θ cells **(A)** THP-1/IL-32θ cells were transfected with 1 μg of pcDNA3.1+ empty vector, pcDNA3.1+-PU.1 and/or C/EBPα expression vectors and incubated overnight. Cells were stimulated with 30 nM PMA for 72 h, and transfection efficiency was confirmed by RT-PCR and Western blot analyses. **(B)** Morphological changes were assessed by phase-contrast microscopy (100 ×). Scale bars represent 20 μm. After washing out non-adherent (undifferentiated) cells with PBS, the adherent cells were stained with Diff-Quick solution. At least 150 stained cells were counted in three randomly selected fields. To assess CD11b **(C)**, CD18 **(D)**, and CD36 **(E)** expression levels, cells were prepared as described above, and mRNA levels were measured by qRT-PCR. Data are presented as mean ± standard error of mean (*n* = 3). *p < 0.05. THP-1/IL-32θ cells versus cells transfected with PU.1 and/or C/EBPα, after PMA treatment.

C/EBPα is a member of the Ets transcription factor family and is a key regulator during myeloid lineage development [40]. Although IL-32θ had no effect on the expression C/EBPα (data not shown), we predicted that increased expression of C/EBPα could support PU.1-mediated cell differentiation. To address this possibility, THP-1/IL-32θ cells were transfected with 1 μg of the C/EBPα and/or PU.1-expressing vectors, and transfection efficiency was confirmed by RT-PCR and Western blot analyses (Figure 7A). Morphological changes were monitored and differentiated cells were again quantified by enumerating adherent cells in culture dishes. The number of differentiated cells was significantly increased by co-transfection with PU.1 and C/EBPα compared to transfection with PU.1 or C/EBPα alone (Figure 7B). The expression levels of CD11b, CD18, and CD36 were also significantly increased in cells co-transfected with PU.1 and C/EBPα (Figure 7C–7E). Meanwhile, transfection with C/EBPα alone, resulted in a significant increase in CD18 expression levels. These findings indicate that overexpression of both PU.1 and C/EBPα resulted in a synergistic effect in the restoration of monocytic differentiation in IL-32θ-expressing cells.

## DISCUSSION

Myeloid cell differentiation is tightly controlled by regulating cytokines and transcription factors because blockade of myeloid lineage development causes blood cancer and immune diseases [41]. Granulocyte-macrophage colony-stimulating factor (GM-CSF) and several interleukins are linked closely to regulate lineage commitment [42–44]. However, most studies on IL-32 have focused on the proinflammatory functions during the innate immune response. Recently, however, the role of IL-32 in apoptosis and metastasis has been investigated. In addition, IL-32 expression has been identified as a marker of gastric cancer [45–48]. Since its initial discovery, studies on relationship between IL-32 expression and cell differentiation are quite rare, but have been steadily published. IL-32 induces the differentiation of monocytes into macrophages through induction of thymic stromal lymphopoietin [49]. Furthermore, it has been reported that IL-32 is a potent regulator of osteoclastogenesis *in vitro* and exhibits a synergistic effect with IL-17 on differentiation of osteoclasts [50–51]. In dendritic cells, it was also known that maturation is mediated by IL-32γ-induced expression of IL-12 and IL-6 [24]. Although IL-32 was shown to enhance cellular differentiation of monocytes into macrophages, intracellular expression of IL-32α repressed differentiation of THP-1 cells by inhibiting PU.1 expression in a STAT3-dependent manner [31]. The roles of other IL-32 isoforms in cell differentiation are unclear, and it seems that the role in myeloid differentiation depends on each isoforms of IL-32. We previously reported that IL-32θ expression attenuates phosphorylation of PU.1, resulting in a reduction of IL-1β production [29]. PU.1, a member of the Ets family of transcription factors, regulates expression of macrophage-specific genes, including CD11b [52], CD18 [53], and the glycoprotein pDP4 [54], and is essential for monocyte differentiation [55–57]. We, therefore, could expect that IL-32θ may decrease PU.1 expression because PU.1 performs auto-regulatory functions, via binding to a distal enhancer on the PU.1 promoter region [58]. Similar to PU.1, C/EBPα is a transcription factor that contributes to the differentiation of monocytes into macrophage [59]. C/EBPα also binds to the distal enhancer of PU.1, thereby inducing PU.1 expression [60]. Additionally, C/EBPα forms heterodimers with AP-1, which induces PU.1 promoter activity more potently than C/EBPα homodimers or AP-1 alone [61].

We, therefore, designed a model for the comparative analysis of monocytic differentiations between IL-32β and IL-32θ in THP-1 cell lines, and predicted that IL-32θ would be involved in regulating monocyte differentiation and that PU.1 and C/EBPα are key components in this regulatory process. In the present study, expression of IL-32θ was found to negatively regulate PMA-mediated differentiation of a monocyte cell line into macrophages compared with IL-32β. IL-32θ expression suppressed morphological changes and the adhesion capability of THP-1 cells to culture plates and to vascular endothelial cells. IL-32θ also inhibited expression of the macrophage markers, CD11b, CD18, and CD36, in THP-1/IL-32θ cells, even after PMA stimulation. Additionally, PU.1 expression levels were decreased in THP-1/IL-32θ cells compared to the wild type and THP-1/IL-32β population. In THP-1/IL-32θ cells, however, overexpression of PU.1 and/or C/EBPα rescued the observed differentiation defect after PMA treatment. Together, these data indicate that IL-32θ is a potent inhibitor of monocytic differentiation and that this inhibition occurs due to a reduction in PU.1 expression. There have been steady attempts to treat myeloid leukemia, called ‘differentiation therapy’ [62–63]. The IL-32θ may be a potent therapeutic target for myeloid differentiation-mediated diseases. In further studies, it will be crucial to examine the effects of IL-32θ expression in IL-32θ transgenic mice or in primary myeloid cells from leukemia patients.

## MATERIALS AND METHODS

### Cell culture and the generation of a cell line stably expressing IL-32β and IL-32θ

The human monocytic cell lines THP-1 and HL-60 were cultured in RPMI 1640 (HyClone, Logan, UT) medium, supplemented with 10% heat inactivated fetal bovine serum, 2 mM l-glutamine, 100 U/mL penicillin, and 100 μg/mL streptomycin (HyClone, Logan, UT). In order to induce differentiation, cells were treated with phorbol 12-myristate 13-acetate (PMA) (St. Louis, MO). The cell line stably expressing IL-32θ and the mock control cell line were previously established and described [29]. To establish constitutive expression of IL-32β, THP-1 cells were transfected with the pcDNA3.1+6xMyc-IL-32β vector, using the Neon™ transfection system (Invitrogen, Carlsbad, CA). Cells were incubated with G-418 (700 μg/mL) and resistant cells were screened for 3 weeks, and expanded clones were acquired by serial dilution.

### Cell morphology and cell adhesion assays

THP-1 cells stably expressing IL-32β and HL-60 cells transfected with IL-32θ were adhered to the bottom of culture wells by treatment with 30 nM and 50 nM of PMA, respectively, for an indicated time, and morphological changes were assessed by phase contrast microscopy at 100× and 200×. Cells were visualized using the Reastain Quick-Diff kit (Reagena, Toivala, Finland). The counting method used was described previously [30]. Briefly, after fixation and staining, a minimum of 150 cells/field were counted in three or more randomly selected fields. To assess the adhesion capability of the cell lines to vascular endothelium, we followed the manufacturer's instructions for the CytoSelect™ Leukocyte-Endothelium Adhesion Assay kit (Cell Biolabs, San Diego, CA, USA), by using HUVEC endothelial cells.

### Construction of expression vectors

We previously identified the IL-32θ isoform in human dendritic cells differentiated by treatment with lipopolysaccharide [20]. To create IL-32θ and PU.1 expression vectors, 6x-myc and HA tags were first inserted into the mammalian expression vector pcDNA 3.1+, generating pcDNA 3.1+/6 × Myc and pcDNA 3.1+/HA. The sequences of IL-32θ and PU.1 were amplified by RT-PCR, digested using *EcoR*I and *Xho*I restriction enzymes, and ligated into their respective vectors, generating pcDNA 3.1+/6 × Myc-IL-32θ and pcDNA 3.1+/HA-PU.1 [29, 31]. The C/EBPα-encoding sequence was subcloned into the pcDNA 3.1+-5 × Flag vector as previously reported [26].

### MTS assay

To examine the effects of IL-32θ and IL-32β expression on cell proliferation, cell viability was evaluated in THP-1 cells by using the CellTiter 96^®^ AQueous One Solution Assay (Promega, Madison, WI, USA). Cells were seeded in 96-well plates, treated with 30 nM of PMA, and incubated for 72 h. Untreated cells were used as controls. AQueous One solution, containing 3-(4,5-dimethylthiazol-2-yl)-5-(3-carboxymethoxyphenyl)-2-(4-sulfophenyl)-2H-tetrazolium (MTS) and phenazine methosulfate (PMS), an electron coupling reagent, was diluted 1:5 in free medium, and 100-μL aliquots of the reagent were added to each well. After 30 min of incubation, the absorbance at 492 nm was measured using an Apollo LB 9110 microplate reader (Berthold Technologies GmbH, Bad Wildbad, Germany).

### Propidium iodide staining

Approximately 1.5 × 10^5^ cells/well were plated in 6-well plates and treated with 30 nM of PMA for 72 h. Cells were then washed twice with PBS and fixed by incubation with 70% ethanol at −20°C. The fixed cells were washed with PBS prior to staining with PBS containing 50 μg/mL PI and 100 μg/mL RNase A, for 30 min in the absence of light. The percentage of PI-stained cells in each cell cycle phase was determined using a FACSCalibur flow cytometer and analyzed with CellQuest Pro software (BD Biosciences, San Jose, CA, USA).

### Quantitative real-time polymerase chain reaction (qRT-PCR) and reverse transcription polymerase chain reaction (RT-PCR) analyses

After PMA treatment, total RNA was extracted from each cell line by using the RNA-BLUE™ total RNA extraction kit (iNtRon Biotechnology, Seoul, Korea), according to the manufacturer's protocol. The cDNA products were prepared using M-MuLV reverse transcriptase (New England Biolabs, Beverly, MA, USA). The mRNA expression levels of the macrophage-specific cell surface markers CD11b, CD18, and CD36 and the transcription factor PU.1 were detected by qRT-PCR, using a relative quantification protocol in a Chromo 4 Real-Time PCR system (Bio-Rad, Hercules, CA, USA) with the SensiFAST™ SYBR^®^ No-ROX Kit (Bioline, Taunton, MA, USA). The CD11b, CD18, and CD36 primers sequences were as follows: CD11b, 5′-TTC CAA GAG AAC GCA AGG GG-3′ (sense) and 5′-TAG TCG CAC TGG TAG AGG CT-3′ (anti-sense); CD18, 5′-TGC TGA TCG GCA TTC TCC TGC TGG TCA TCT-3′ (sense) and 5′-CAC TGG GAC TTG AGC TTC TCC TTC TCA AAG-3′ (anti-sense); CD36, 5′-CTG GCT GTG TTT GGA GGT AT-3′ (sense) and 5′-TCT GTG CCT GTT TTA ACCCA-3′ (anti-sense); PU.1, 5′-CTG GCT GTG TTT GGA GGT AT-3′ (sense) and 5′-TCT GTG CCT GTT TTA ACC CA-3′ (anti-sense). For confirmation of transient transfection, RT-PCR was performed using IL-32-, PU.1-, and C/EBPα-specific primers. The primer sequences were as follows: IL-32, 5′-CTG GCT GTG TTT GGA GGT AT-3′ (sense) and 5′-TCT GTG CCT GTT TTA ACC CA-3′ (anti-sense); PU.1, 5′-ATG TTA CAG GCG TGC AAA ATG-3′ (sense) and 5′-TGC TTG GAC GAG AAC TGG AA-3′ (anti-sense); C/EBPα, 5′-ACG AGA CGT CCA TCG ACA TC-3′ (sense) and 5′-CAG TGC GCG ATC TGG AAC TG-3′ (anti-sense); GAPDH, 5′-GGC TGC TTT TAA CTC TGG TA-3′ (sense) and 5′-TGG AAG ATG GTG ATG GGA TT-3′ (anti-sense). GAPDH was used as an internal control.

### Flow cytometry analyses

To determine the effect of IL-32θ on the expression of the macrophage surface markers CD18 and CD36, untreated THP-1 cells and cells stimulated with PMA were assessed by flow cytometry. Cells were stained with saturated concentration of anti-CD36 (Nordic-MUbio, Susteren, Netherland) or anti-CD18 (Millipore, Ma, USA) mAbs for 1 h in PBS containing 1% BSA. Cells were then washed and incubated with a FITC-conjugated goat anti-mouse antibody for 1 h. The expression levels of CD18 and CD36 were measured using a FACS Calibur flow cytometer and analyzed with CellQuest Pro software.

### Western blot analyses

Cells were harvested and lysed with 50 mM HEPES (pH 7.5), 150 mM NaCl, 5% glycerol, 20 mM β-glycerophosphate, 1% Nonidet P-40, 0.5% Triton X-100, 1 mM EDTA, and 1 mM EGTA. Western blotting was performed using primary antibodies specific for cell cycle analysis; cyclin D, cyclin E, p27 from Santa Cruz Biotechnology (Santa Cruz, CA, USA), and anti-C/EBPα antibody and HRP-conjugated secondary antibodies from Millipore (Billerica, MA, USA). KU32–52, a monoclonal anti-IL-32 antibody, was produced as previously reported [32].

### Statistical analysis

Quantitative data presented in figures represent the mean ± SEM of results from at least 3 independent experiments. Statistical significance for multiple groups was assessed using one-way ANOVA, followed by Tukey's HSD tests. *p < 0.05 was considered statistically significant.
